# Diagnosis of Pancreatic Solid Lesions, Subepithelial Lesions, and Lymph Nodes Using Endoscopic Ultrasound

**DOI:** 10.3390/jcm10051076

**Published:** 2021-03-05

**Authors:** Akashi Fujita, Shomei Ryozawa, Masafumi Mizuide, Yuki Tanisaka, Tomoya Ogawa, Masahiro Suzuki, Hiromune Katsuda, Yoichi Saito, Tomoaki Tashima, Kazuya Miyaguchi, Eiichi Arai, Tomonori Kawasaki, Yumi Mashimo

**Affiliations:** 1 Department of Gastroenterology, Saitama Medical University International Medical Center, 1397-1, Yamane, Hidaka, Saitama 350-1298, Japan; a.fujita0628@gmail.com (A.F.); mizuide1971@yahoo.co.jp (M.M.); tanisaka1205@gmail.com (Y.T.); t.ogawa0210@icloud.com (T.O.); msuzgast@tmd.ac.jp (M.S.); hk0112@saitama-med.ac.jp (H.K.); stm_ys41@yahoo.co.jp (Y.S.); tomo3029@saitama-med.ac.jp (T.T.); kaz.hr77@gmail.com (K.M.); ymashimo@saitama-med.ac.jp (Y.M.); 2 Department of Pathology, Saitama Medical University International Medical Center, 1397-1, Yamane, Hidaka, Saitama 350-1298, Japan; e_arai@saitama-med.ac.jp (E.A.); tomo.kawasaki.14@gmail.com (T.K.)

**Keywords:** endoscopic ultrasound, endoscopic ultrasound-guided fine-needle aspiration, fine-needle biopsy, diagnostic accuracy, pancreatic solid lesions, subepithelial lesions, lymph nodes, lymphadenopathy

## Abstract

Currently, endoscopic ultrasound (EUS) has become widely accepted and has considerable advantages over computed tomography (CT) and other imaging modalities, given that it enables echostructure assessment in lesions with <1 cm diameter and permits high resolution imaging. EUS-guided tissue acquisition (EUS-TA) provides consistent results under ultrasound guidance and has been considered more effective compared to CT- or ultrasound-guided lesion biopsy. Moreover, complication rates, including pancreatitis and bleeding, have been extremely low, with <1% morbidity and mortality rates, thereby suggesting the exceptional overall safety of EUS-TA. The aggressive use of EUS for various lesions has been key in facilitating early diagnosis and therapy. This review summarizes the diagnostic ability of EUS for pancreatic solid lesions, subepithelial lesions, and lymph nodes where it is mainly used. EUS has played an important role in diagnosing these lesions and planning treatment strategies. Future developments in EUS imaging technology, such as producing images close to histopathological findings, are expected to further improve its diagnostic ability. Moreover, tissue acquisition via EUS is expected to be used for precision medicine, which facilitates the selection of an appropriate therapeutic agent by increasing the amount of tissue collected and improving genetic analysis.

## 1. Introduction

Currently, endoscopic ultrasound (EUS) has become widely accepted for evaluating pancreatobiliary diseases and other abdominal tumors. It offers considerable advantages over computed tomography (CT) and other imaging modalities given that it enables echostructure assessment in lesions with <1 cm diameter and permits high resolution imaging. Apart from purely diagnostic imaging, EUS has progressed to tissue acquisition and therapeutic procedures [1]. Two differently shaped EUS scopes have been developed, namely radial and linear arrays. Accordingly, radial EUS has a viewing angle of 360 degrees, which can help differentiate between the lesion and organs around it that may appear similar to the lesion. On the other hand, the advantage of the linear array echoendoscope lies in its ability to be used for tissue acquisition through endoscopic ultrasound-guided tissue acquisition (EUS-TA) [2].

EUS-TA allows for obtaining material from abnormal lesions via the gastrointestinal wall for tissue analysis [3]. First reported in 1992 [4], EUS-TA has been well established worldwide. This widely used tissue sampling procedure provides consistent results under ultrasound guidance [5,6,7] and has been considered more effective compared to CT- or ultrasound-guided lesion biopsy [8]. Moreover, complication rates, including pancreatitis and bleeding, have been extremely low, with <1% morbidity and mortality rates, suggesting its impeccable safety [9,10]. EUS-TA has been mainly used for pancreatic solid lesions [11,12], abdominal or mediastinal lymph nodes [13,14,15], and gastrointestinal subepithelial lesions (SELs) [7,16], with other indications including liver lesions [17], adrenal grand lesions [18], and biliary strictures [19,20]. However, indications for pancreatic cystic lesions have varied significantly between countries due to the risk of dissemination [21,22].

The aggressive use of EUS for various lesions has been key in facilitating early diagnosis and therapy given that it helps distinguish between benign and malignant tumors when determining whether surgery or follow-up is needed, diagnose the degree of malignant tumor progression when undetermined lymph node swelling is detected, and obtain histological evidence of cancer when chemotherapy is selected [23].

EUS is expected to play an increasingly important role in improving prognosis through early diagnosis, especially for abdominal tumors. Therefore, the current review focuses on the diagnostic ability of EUS for pancreatic solid lesions, SELs, and lymph nodes where it is mainly used.

## 2. Pancreatic Solid Lesions

**Figure 1:**
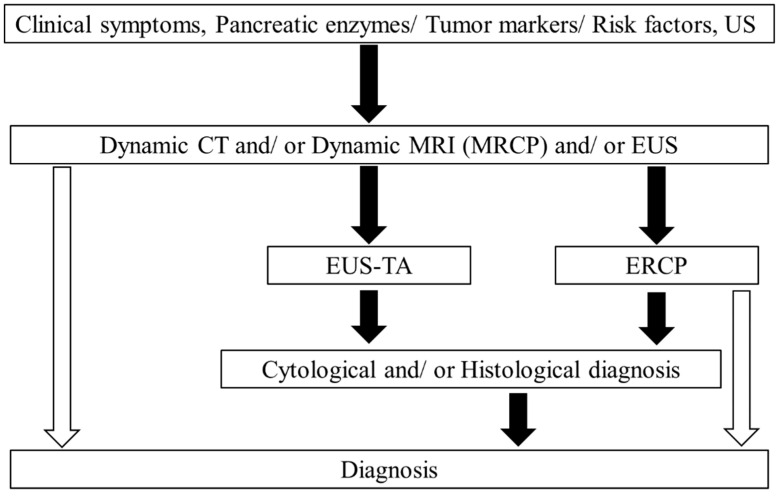
Algorithm for pancreatic cancer diagnosis (from [26]). Black arrows indicate higher performance frequency, whereas white arrows indicate lower performance frequency. US, ultrasound; CT, computed tomography; MRI, magnetic resonance imaging; MRCP, magnetic resonance cholangiopancreatography; EUS, endoscopic ultrasound; ERCP, endoscopic retrograde cholangiopancreatography; EUS-TA, endoscopic ultrasound-guided tissue acquisition.

To detect pancreatic cancer, several imaging modalities, including ultrasonography (US), EUS, CT, magnetic resonance imaging (MRI), endoscopic retrograde cholangiopancreatography (ERCP), and 18F-fluorodeoxyglucose positron emission tomography (PET) have been used. Most pancreatic cancer cases develop main pancreatic duct stenosis at the lesion site and distal dilatation, which is often accompanied by focal branch duct dilatation and cyst formation adjacent to the tumor. Cases with carcinoma in situ may exhibit irregularities in the pancreatic duct diameter due to localized pancreatic duct stenosis or hypoechoic changes reflecting obstructive pancreatitis caused by stenosis. Studies have shown the usefulness of EUS given that other imaging modalities often fail to detect small lesions [24]. Tumor detection rates for early stage of pancreatic cancer (Stage 0, I) have been reported to be 76.3%, 51.5%, 45.1%, and 52.6% using EUS, CT, MRI, and US, respectively [25]. Considering this situation, recent guidelines suggest performing EUS, as well as CT or MRI, upon diagnosis [26]. The recommended diagnostic algorism is shown in Figure 1.

**Figure 2:**
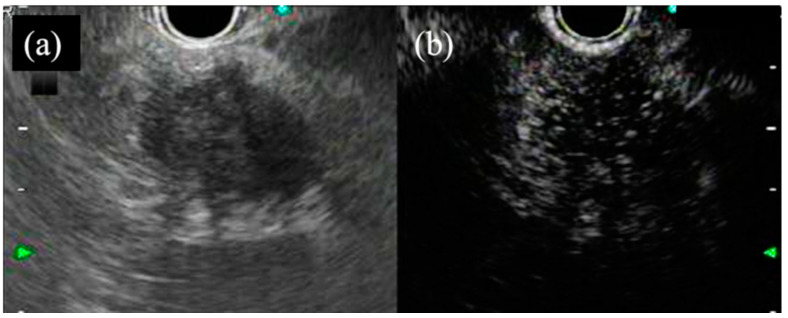
A typical example of pancreatic adenocarcinoma with hypoenhancement. Fundamental B-mode endoscopic ultrasound (EUS) (**a**) showing a hypoechoic tumor at the pancreatic body, and contrast-harmonic EUS (**b**) showing the tumor with hypoenhancement.

**Figure 3:**
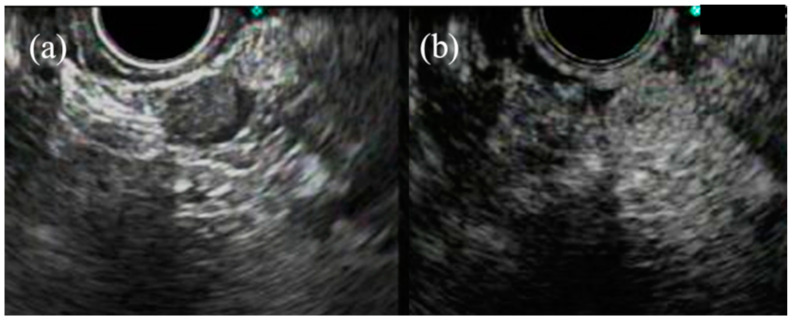
A typical example of a pancreatic neuroendocrine tumor with hyperenhancement. Fundamental B-mode endoscopic ultrasound (EUS) (**a**) showing a hypo-isoechoic tumor at the pancreatic head, and contrast-harmonic EUS (**b**) showing a tumor with hyperenhancement.

Additionally, studies have suggested the utility of contrast-harmonic EUS (CH-EUS), which allows for the evaluation of pancreatic lesion vascularity. Contrast harmonic imaging allows real-time depiction of microvessels and parenchymal perfusion without doppler-related artifacts [27]. Pancreatic solid lesions can be classified into four categories: nonenhancement, hypoenhancement, isoenhancement, and hyperenhancement lesions (Figure 2 and Figure 3). Kitano et al. reported that a hypoenhancement pattern, determined via CH-EUS, had a sensitivity and specificity of 95.1% and 89.0% for diagnosing ductal carcinomas, while a hyperenhancement pattern had a sensitivity and specificity of 78.9% and 98.0% for diagnosing neuroendocrine tumors, respectively [28]. A recent meta-analysis on the utility of CH-EUS with enhancement pattern assessment showed a pooled sensitivity and specificity of 93% and 80%, respectively, for the diagnosis of pancreatic cancer [27]. Moreover, CH-EUS can be advantageous for patients who have contraindications to MRI and CT contrast agents, such as renal failure or contrast allergies. CH-EUS also allows for dynamic and repeat examinations, given that it does not expose patients to ionizing radiation [29]. Considering the aforementioned reasons, CH-EUS indeed plays an important role in clinical practice and can be certainly expected to be developed into a modality that approaches pathological diagnosis in the near future.

Apart from diagnostic imaging, EUS-TA can be useful in the differential diagnosis of pancreatic solid lesions, with good diagnostic performance having been reported. Five meta-analyses have reported that endoscopic ultrasound-guided fine-needle aspiration (FNA) and fine-needle biopsy (FNB) have sensitivities and specificities of 84–92% and 96–98%, respectively, [30,31,32,33,34] (Table 1). Several factors can affect the outcome of this technique, with evidence showing that rapid onsite evaluation (ROSE) increases the diagnostic performance of EUS-TA [32,35]. A randomized trial compared standard EUS-TA with CH-EUS guided TA. There was no significant difference in diagnostic performance between these groups [36]. However, CH-EUS can help to identify the target for EUS-TA, with easier avoidance of necrosis described as nonenhancement areas and vessels inside the tumor [28]. Needle selection has been considered an essential factor and has consequently been evaluated in numerous studies. Although the use of an FNA needle was traditionally considered first-line, the FNB needle is increasingly more common in clinical practice to improve yield. A randomized crossover study showed statistically significant differences in sensitivity (82% vs. 71%) and accuracy (84% vs. 75%) between FNB and FNA needles, respectively [37]. Recently, two different FNB needles have been mainly used, including the fork-tip needle (SharkCore; Medtronic, Newton, Mass and Covidien, Dublin, Ireland), which is characterized by two sharp tips on the opposite side of the lumen [38], and the franseen-type needle (Acquire; Boston Scientific, Marlborough, MA, USA), which is characterized by three symmetric cutting tips [39]. A meta-analysis comparing the two needles for the EUS-FNB of solid mass lesions was published. The analysis featured a total of 21 studies with 1632 patients. The pooled diagnostic yield with fork-tip needle was 92.8% (95% CI 85.3–96.6, *I*2 = 73.1), whereas the pooled diagnostic yield using the franseen needle was 92.7% (95% CI 86.4–96.2, *I*2 = 88.4), demonstrating no statistical difference between the needles (*p* = 0.98). These needles provided a higher rate of extremely good-quality histologic samples and required fewer needle passes to reach a diagnosis compared to other FNB needles [40]. A recent randomized trial comparing EUS-FNA+ROSE with EUS-FNB alone demonstrated equal diagnostic yield [41] Thus, in medical centers where ROSE has not been applicable, FNB may still be an effective option. However, no definitive recommendations can be made in favor of using one particular device given no strong diagnostic superiority of one needle over another [42,43,44,45,46]. Thus, endoscopists are encouraged to select the appropriate needle for the situation. A 25G needle may be better than a 22G or 19G one for lesions difficult to puncture, while an FNB needle may provide further information on tissue architecture, as well as a greater sample yield, which would allow for further analyses, such as genetic sequencing and phenotyping. This again may enable more personalized treatment strategies [23].

Sometimes it is difficult to perform EUS-TA for early stage of pancreatic cancer. In such cases, ERCP may be useful and the sensitivity of serial pancreatic juice cytology by ERCP is reported to be 77.2–100%. However, the incidence of acute pancreatitis due to diagnostic ERCP is reported to be 0.7–11.8% [47]. Therefore, the indication of ERCP needs to be considered carefully.

Although EUS-TA is exceedingly safe and useful for diagnosing pancreatic solid lesions, needle tract seeding remains a concern for preoperative cases [48]. Recently, neoadjuvant therapy has been found to facilitate the possibility of surgery in borderline resectable pancreatic cancer [49,50,51]. In a randomized controlled trial comparing neoadjuvant chemotherapy with gemcitabine administration and S-1 with upfront surgery, neoadjuvant chemotherapy demonstrated to have considerable survival benefits for patients with resectable pancreatic cancer [52]. Therefore, obtaining an accurate preoperative diagnosis is especially important. Although needle tract seeding is an extremely rare adverse event and has mostly been observed in patients with pancreatic adenocarcinoma, it can also occur after solid pseudopapillary neoplasms [53]. Despite the unclear developmental process of needle tract seeding, certain measures should be established to reduce the risk of needle tract seeding for preoperative pancreatic solid lesions.

## 3. Subepithelial Lesions

SELs are often found incidentally upon esophagogastroduodenoscopy [54]. Although extramural compressions are mostly caused by normal organs, such as the spleen or splenic artery, pancreas, gallbladder, heart, and the left lobe of the liver, they can also be caused by pathologic structures, such as pseudocysts, an enlarged gallbladder, and splenic artery aneurysms or tumors [55,56]. Several SELs are benign, such as lipomas, ectopic pancreas, leiomyomas, schwannomas, or lymphangioleiomyoma. However, up to 13% of upper gastrointestinal tract lesions are malignant, such as metastatic or malignant lymphomas, while an additional 8% have at least a malignant potential, such as gastrointestinal stromal tumors (GIST) [57]. Considering the difficulty of distinguishing between malignant and non-malignant tumors through endoscopic appearance alone, further characterization and management of these lesions through other modalities is important.

Conventional endoscopic forceps biopsy is limited because these forceps usually cannot reach the tumor. The diagnostic yield of the bite-on-bite technique, in which each bite is directly on top of the previous bite to burrow into the lesion, is poor, ranging from 17% to 58.9% [58,59]. Through EUS, SELs can be diagnosed through the evaluation of their originating layer, echo level, and internal echo pattern. The gastrointestinal wall can be viewed as a five-layer structure with lower frequency (7.5–12 MHz) [60]. The EUS layer, location within the gastrointestinal tract, and echo features can provide valuable information with which a possible diagnosis can be established. Regarding the echo features, hyperechoic and anechoic lesions can be initially differentiated from hypoechoic, isoechoic, or mixed echogenic lesions. Hyperechoic lesions are generally benign and most often indicate lipomas, with no further work-up being needed when no mixed features are found within a hyperechoic lesion [61]. Anechoic lesions are fluid-filled structures that can indicate vascular lesions (e.g., varices) or cystic lesions (e.g., lymphangioma), both of which can be easily distinguished by a positive or negative doppler signal, respectively. Mixed lesions with partially solid appearing components require further work-up by other modalities, given that such lesions can indicate solid lesions with cystic degeneration, complicated cystic lesions, or intra-abdominal abscesses [62,63,64]. For hypoechoic, isoechoic, or mixed SELs, specific diagnosis is required considering their possible malignant potential [65,66]. Typical EUS features of SELs are summarized in Table 2.

The differential diagnosis of malignant or potentially malignant SELs from benign lesions is important in determining treatment strategy [67,68]. GIST may require a definite diagnosis before providing treatment interventions, such as surgery or chemotherapy, whereas benign lesions, such as leiomyomas and schwannomas, can often be followed up. Approximately 10% to 30% of GISTs have a malignant clinical course [69,70,71]. Additionally, reports have shown that large GISTs with a high mitotic index frequently exhibit a malignant clinical course, whereas small GISTs with a low mitotic index may also show a malignant course with metastasis [72,73]. However, several SELs, including GIST, with similar echo features originate from the fourth layer, making it difficult to distinguish GIST from other SELs through EUS imaging alone [60].

**Figure 4:**
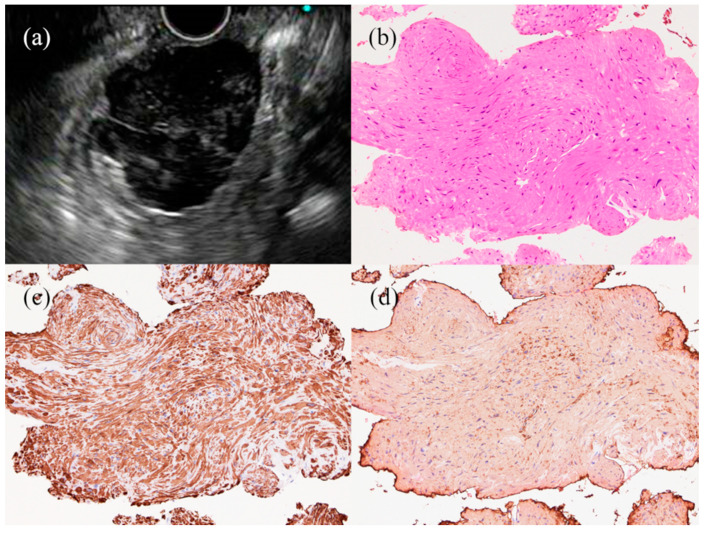
Case of gastric leiomyoma. Endoscopic ultrasound (**a**) showing a hypoechoic tumor originating from the fourth layer. Histopathology (**b**) showing eosinophilic spindle cells with desmin (**c**) and α-SMA (**d**) immuno-expressions on a specimen of endoscopic ultrasound-guided tissue acquisition.

**Figure 5:**
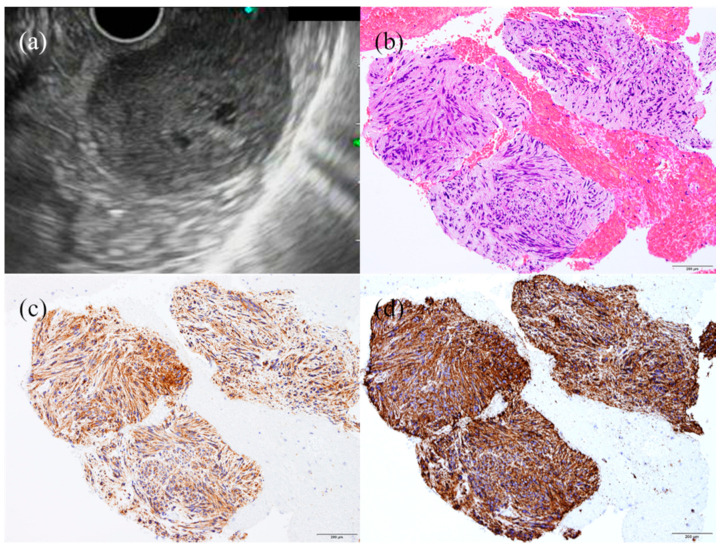
Case of gastric gastrointestinal stromal tumor. Endoscopic ultrasonography (**a**) showing a hypoechoic tumor with a heterogeneous echotexture. Histology (**b**) showing relatively basophilic spindle cells with KIT (**c**) and CD34 (**d**) positivity on a specimen of endoscopic ultrasound-guided tissue acquisition.

GIST is diagnosed through KIT or CD34 positivity following immunohistochemical analysis of the tissue. Tumors negative for KIT, CD34, desmin, and S-100 may require additional tests, including DOG1 staining or the identification of mutations in the KIT or PDGFRA gene [74]. Thus, acquiring tissue samples for immunohistochemistry staining is essential [75] (Figure 4 and Figure 5). EUS-TA has been found to be a useful, minimally invasive procedure for tissue acquisition from SELs, with reported diagnostic accuracy rates reigning from 52% to 92% [16,76,77,78,79,80]. Moreover, a recent report revealed a diagnostic accuracy of 87.5% of a forward-viewing echoendoscope even for small lesions (mean lesion diameter of 10.6 mm) [81].

Apart from EUS-TA, other endoscopic tissue acquisition techniques have been recently reported and clinically applied to obtain more SEL tissue volumes. The reported diagnostic rates of various endoscopic tissue-obtaining methods using endoscopic submucosal dissection (ESD) techniques or endoscopic snare resection techniques have ranged from 85% to 94% [82,83]. However, ESD and the endoscopic snare resection technique have disadvantages. Given the invasive nature of these procedures, endoscopists should pay special attention to intraoperative bleeding and perforation due to risk for severe hypotension or tumor cell seeding as a consequence thereof [84]. Reports have shown that minor complications (procedural-relate oozing) occurred in 56% of patients who underwent endoscopic partial removal using the unroofing technique, although no severe complication had occurred [83]. Furthermore, using the aforementioned procedures for tissue sampling of SELs with an extraluminal growth pattern is difficult [82]. In contrast, EUS-TA is safe and reliable while being unmatched in its ability to distinguish between different types of SELs, especially those originating from the fourth EUS layer [7,85].

## 4. Lymph Nodes

**Figure 6:**
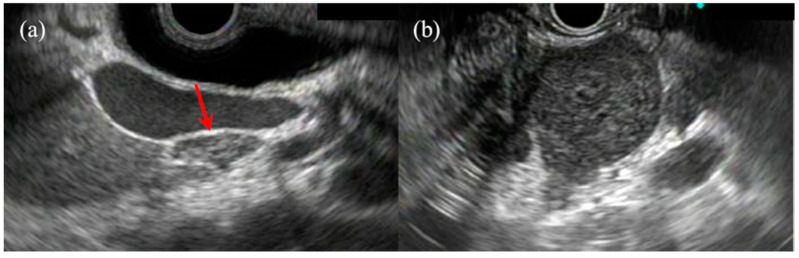
(**a**) A typical finding for a benign lymph node (LN). This lesion shows flat cross-sections, internal hyperechoic features, and <10 mm diameter (red arrow). (**b**) A typical finding for a malignant LN. This lesion shows round cross-sections, sharp demarcations, internal hypoechoic features, and 25 mm diameter.

The distinction between benign and malignant lymph nodes is particularly important when planning for the treatment of various diseases. Although other imaging modalities, such as CT and PET, can detect enlarged lymph nodes, they lack sufficient accuracy to distinguish benign from malignant lymphadenopathies [86,87]. Traditional thoracotomy, thoracoscopy, and laparoscopy, which can accurately establish a pathological diagnosis, will be invasive for patients with benign lymphadenopathy [88]. On the other hand, EUS can effectively detect and evaluate mediastinal and abdominal lymph nodes. The proposed EUS-based diagnostic criteria for malignant lymphadenopathies include round or oval cross-sections, sharp demarcations, internal hypoechoic features, and >10 mm largest diameter (Figure 6). Overall, EUS alone has a diagnostic accuracy of 80% when all criteria are met [89]. Therefore, distinguishing between benign and malignancy and determining the most appropriate cancer management using EUS imaging of lymphadenopathy alone remains challenging [89,90].

EUS-TA is useful when other modalities, including EUS, are unreliable. Already a part of general practice, EUS-TA for lymphadenopathy helps identify malignancy and diagnose inflammatory diseases, including tuberculosis [91,92,93]. The advantages of EUS-TA for the lymph nodes include: (1) staging of malignant diseases (N-staging), (2) identification of an unknown primary tumor or clarifying which cancer has spread to the lymph nodes when multiple cancers are present (primary identification), (3) diagnosis of recurrence when enlarged lymph nodes appear following cancer surgery, and (4) puncturing the lymph nodes to obtain a sample for histological diagnosis in cases where puncturing the primary tumor is difficult. EUS-TA of the lymph nodes may change the diagnosis and treatment plan [94]. EUS-TA is indicated for mediastinal lymph nodes that can be punctured from the esophagus, abdominal lymph nodes from the stomach and duodenum, and pelvic lymph nodes from the rectum. Attempts at describing abdominal lymph nodes through EUS is particularly important given that gastrointestinal tract stretching using scope manipulation may facilitate the visualization of puncturable lesions that appear to be distant from the stomach or duodenum on CT images or other puncturable lymph nodes. Advance understanding of the positional relationship between the lymph nodes to be punctured and the surrounding vessels and organs based on the findings of CT and PET is imperative.

A meta-analysis reported that EUS-TA had a pooled sensitivity, specificity, positive likelihood ratio, and negative likelihood ratio of 87% (95% confidence interval (CI) 86–90%), 100% (95% CI 99–100%), 68.98 (95% CI 42.10–113.02), and 0.14 (95% CI 0.11–0.17) in the differential diagnosis of benign and malignant lymph nodes, respectively [95]. EUS-TA has also been found useful in diagnosing malignant lymphoma. In fact, some studies have reported high diagnostic yields using flow cytometry for malignant lymphomas [96,97], with the selective use of flow cytometry potentially improving diagnostic outcomes [98]. Yasuda et al. [6] were able to subtype, according to the World Health Organization classification, 44 of their 48 patients with lymphoma who subsequently received multiple tailored treatments, including chemotherapy. They used a 19G needle to safely perform EUS-TA, with only 1% of their patients developing complications. To increase the rate of subtyping, using 19-gauge needle may be useful.

In case of lower gastrointestinal tract approach for pelvic lymphadenopathy, EUS-TA has been reported to be useful for urological cancer types, including prostate and bladder cancer, with a sensitivity of 94.4% [99]. Therefore, EUS-TA has been widely applied in diagnosing lymph nodes.

## 5. Conclusions

This review summarized the diagnostic ability of EUS for pancreatic solid lesions, SELs, and lymph nodes where it is mainly used. EUS has played an important role in diagnosing these lesions and planning treatment strategies. Future developments in EUS imaging technology, such as producing images close to histopathological findings, can be expected to further improve its diagnostic ability. Moreover, tissue acquisition via EUS is expected to be used for precision medicine, which would facilitate the selection of therapeutic agents by increasing the amount of tissue collected and improving genetic analysis.

## Figures and Tables

**Table 1:** Meta-analyses of endoscopic ultrasound-guided fine-needle aspiration or fine-needle biopsy for solid pancreatic lesions.

Reference	Year	Cases (n)	Sensitivity	Specificity
Hewitt [32]	2012	FNA: 4984	85	98
Chen [31]	2012	FNA: 1860	92	96
Puli [33]	2013	FNA: 4766	86.8	95.8
Banafea [30]	2016	FNA: 2761	90.8	96.5
Yang [34]	2016	FNB: 828	84	98

**Table 2:** Typical features of subepithelial lesions (from [60,61]).

SELs	EUS Layer	EUS Imaging Feature	Histology	Malignant Potential
Leiomyoma	2nd or 4th	Hypoechoic (iso- or hypoechoic compared to muscle layer), homogeneous, sometimes calcifications	Desmin (+), α-SMA (+)	None (primary leiomyosarcoma: extremely rare)
Schwannoma	3rd or 4th	Hypoechoic, round or oval, homogeneous, well-demarcated	S-100 (+)	Extremely rare
Ectopic pancreas	3rd (and 4th)	Hypoechoic, or mixed echogenicity heterogeneous echotexture, umbilication, ductal structures, indistinct margins	Pancreatic tissue	Extremely rare
Lipoma	3rd	Hyperechoic, smooth margins, homogeneous, may be polypoid	Mature lipocytes	None
Brunnerioma	3rd	Hyperechoic, smooth margin, possibly hypoechoic-dilated gland duct	Hyperplasia of the Brunner gland	None
Lymphangioma	3rd	Anechoic, occasionally multiloculated	No solid components	None
Varices	2nd or 3rd	Anechoic, serpiginous structure with doppler signal	No solid components	None
Granular cell tumor	2nd, 3rd, or 4th	Hypo- or isoechoic, oval, homogeneous, smooth margins	PAS (+), S-100 (+), and NSE (+)	Extremely low risk of malignancy (2–4%)
Glomus tumor	3rd or 4th	Round, hypoechoic, homogeneous, may have a halo	α-SMA (+), vimentin (+), laminin (+), CD34 (rarely), and KIT (-)	Rare
GIST	4th	Benign features: small (≤2 cm), oval or round, hypoechoic but relatively hyperechoic compared to muscle layer, homogeneous Malignant features: large (>3 cm), irregular margins, heterogeneous echotexture, cystic spaces, hypervascularity, marginal halo, hyperechoic spots/echogenic foci	KIT (+), CD34 (+), desmin (+), S-100 (-), DOG1 (+), or a mutation search of the KIT or PDGFRA gene	10–30% clinically malignant
NET	2nd or 3rd	Oval to round, hypo- or isoechoic, homogeneous, regular margins	Synaptophysin (+), chromogranin (+), INSM1 (+)	Depending on type, size, and location
Lymphoma	2nd, 3rd, or 4th	Hypoechoic	Atypical lymphocyte	Always
metastasis	Any layer	Heterogeneous or hypoechoic	Depending on a primary	Always

## Data Availability

Not applicable.
